# Bovine pericardial extracellular matrix niche modulates human aortic endothelial cell phenotype and function

**DOI:** 10.1038/s41598-019-53230-1

**Published:** 2019-11-13

**Authors:** Jeny Shklover, James McMasters, Alba Alfonso-Garcia, Manuela Lopera Higuita, Alyssa Panitch, Laura Marcu, Leigh Griffiths

**Affiliations:** 1 Present Address: Department of Chemical Engineering, Israel Institute of Technology, Haifa, 31096 Israel; 2 Department of Biomedical Engineering, University of California Davis, One Shields Avenue, Davis, CA 95616 United States; 3 Department of Cardiovascular Diseases, Mayo Clinic, 200 First St. SW, Rochester, MN 55905 United States

**Keywords:** Biotechnology, Cell biology

## Abstract

Xenogeneic biomaterials contain biologically relevant extracellular matrix (ECM) composition and organization, making them potentially ideal surgical grafts and tissue engineering scaffolds. Defining the effect of ECM niche (e.g., basement membrane vs. non-basement membrane) on repopulating cell phenotype and function has important implications for use of xenogeneic biomaterials, particularly in vascular applications. We aim to understand how serous (i.e., basement membrane) versus fibrous (i.e., non-basement membrane) ECM niche of antigen-removed bovine pericardium (AR-BP) scaffolds influence human aortic endothelial cell (hAEC) adhesion, growth, phenotype, inflammatory response and laminin production. At low and moderate seeding densities hAEC proliferation was significantly increased on the serous side. Similarly, ECM niche modulated cellular morphology, with serous side seeding resulting in a more rounded aspect ratio and intact endothelial layer formation. At moderate seeding densities, hAEC production of human laminin was enhanced following serous seeding. Finally, inflammatory marker and pro-inflammatory cytokine expression decreased following long-term cell growth regardless of seeding side. This work demonstrates that at low and moderate seeding densities AR-BP sidedness significantly impacts endothelial cell growth, morphology, human laminin production, and inflammatory state. These findings suggest that ECM niche has a role in modulating response of repopulating recipient cells toward AR-BP scaffolds for vascular applications.

## Introduction

Xenogeneic biomaterials have found increasing clinical utility as surgical patches in various anatomical sites. Bovine pericardium (BP) for instance has been employed in cardiovascular applications such as angioplasty during carotid endarterectomy^1^, valve reconstruction^2^ or congenital intracardiac repair^3,4^. In order to mitigate complications related to immune-rejection, BP patches are typically subjected to glutaraldehyde (GTA) fixation. Unfortunately, although this process reduces recipient acute graft-specific adaptive immune response, it fails to eliminate immunogenicity and chronic recipient immune responses persist^5^. Furthermore, GTA fixation chemically alters the biomaterial’s composition and liberates toxic aldehyde residues^6,7^, limiting repopulating cell viability, endothelialization^8,9^ and recipient cell-mediated remodeling^10,11^. These limitations of GTA fixation result in biomaterial related complications including graft calcification and stenosis^2,12^. Unfixed BP extracellular matrices (ECM) in which antigenicity has been reduced or eliminated have potential to overcome the limitations of current clinically-utilized glutaraldehyde-fixed biomaterials, presenting recipients with a native ECM environment that does not trigger immune recognition and fostering recipient cellular repopulation, integration with host tissues and ultimately matrix remodeling.

An ideal ECM scaffold should ameliorate biomaterial antigenicity, while maintaining native ECM structure-function properties and recellularization capacity. Indeed, the decellularization paradigm was originally conceived as a method to reduce xenogeneic tissue antigenicity, while maintaining native ECM properties^13^. However, persistence of antigens in apparently acellular ECM scaffolds^14–16^, reduced graft durability^17^, and limited preservation of the native ECM^13,18^ have brought into question the viability of decellularization as the primary outcome measure in ECM scaffold production. Furthermore, previous decellularization attempts have been shown to result in varying degrees of ECM damage and universally result in destruction of the integrity of delicate basement membranes^19^. The antigen removal (AR) paradigm has been proposed as a method for specific assessment of residual ECM scaffold antigen content^20^. In support of the AR approach, specific assessment of *in vitro* ECM scaffold antigen content has been shown to correlate with reduction in recipient *in vivo* graft-specific adaptive immune response^21,22^. Specifically, BP scaffolds subjected to sequential removal of hydrophilic and lipophilic antigens using amidosulfobetaine-14 (ASB-14) demonstrate reduced immunogenicity, fostering recipient adaptive immune tolerance, while preservation of native scaffold ECM properties modulates innate immune pro-regenerative integration^17,23,24^. Despite these findings, the impact of native ECM niche preservation, specifically the presence of a basement membrane, in AR ECM scaffolds on the process of endothelialization and maintenance of a healthy endothelial phenotype remains largely unexplored.

Endothelialization is a key factor in modulating recipient response towards ECM scaffolds implanted in cardiovascular sites. Lineage tracking studies demonstrate that following *in vivo* implantation of acellular ECM scaffolds, cellular repopulation occurs predominantly via adhesion of mesenchymal and endothelial precursors from the vascular lumen^25,26^. Forming of an endothelial monolayer takes several weeks following implantation of an acellular ECM scaffold^25^. Complete endothelialization is associated with reduced incidence of thrombosis and calcification^27^, making rapid formation of a quiescent endothelial monolayer a primary concern for the development of tissue engineered scaffolds. Similarly, in valvular applications, *in vitro* endothelialization of xenografts prior to implantation improves ultimate *in vivo* endothelial coverage^28^, valve durability and reduces tissue degeneration^29,30^. However, endothelial cells (EC) can exhibit a normal, or dysfunctional state, with the latter often accompanying inflammation and vessel thickening^31^. Therefore, characterization of endothelial phenotype and function following *in vitro* seeding is key to understanding the likely *in vivo* response to the material upon implantation. The initial cellular response is critical to the healing process, increasing the importance of understanding the impact that ECM niche of seeded AR scaffolds has on repopulating endothelial cell phenotype and function.

The anisotropic organization of BP, which consists of a serous side containing a specialized basement membrane, inferred by specific structural proteins such as laminin and type IV collagen (Col IV), and a fibrous side that exhibits loose collagenous tissue (i.e., type I collagen), allows for investigation into how different ECM niches (i.e., presence or absences of a basement membrane) modulate repopulating EC phenotype and function. We hypothesize that the absence of a basement membrane has the potential to negatively impact the endothelialization of antigen-removed bovine pericardium (AR-BP) scaffolds. In this work we investigate the cellular toxicity of the AR procedure and the effect that AR-BP scaffold sidedness has on human aortic endothelial cell (hAEC) adhesion, growth, human laminin production, and pro-inflammatory cytokine release.

## Results

### Scaffold washing eliminates toxic ASB-14 from AR-BP

We first investigated the sensitivity of hAEC to the ASB-14 utilized in the AR process (Supplemental Fig. S1). The concentration of ASB-14 which was lethal to 50% of hAEC (LD50) was 0.0021% w/v. Analysis of the scaffold decellularization washout solution over the course of 6 days of washing demonstrated a decrease in toxicity with increasing number of washing days (p < 0.0001); and after 6 days of washing, toxicity of components leaching from the scaffold had reached zero (100% cell viability). Although toxicity had reduced dramatically by day 4, with 92.5% cell viability towards wash buffer contents, hAEC adhesion and/or proliferation on the scaffold were still inhibited. After 6 days of washing hAEC adhesion and proliferation on the scaffold reached control levels (Supplemental Fig. S2) and consequently 6 days of scaffold washing was used for all subsequent experiments.

### ECM niche modulates hAEC proliferation but not cellular adhesion

**Figure 1:**
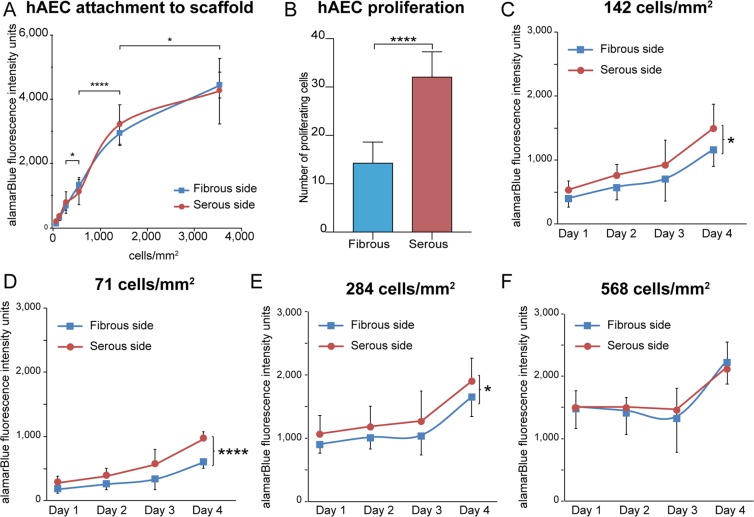
AR-BP sidedness impacts endothelial proliferation but not adhesion. (**A**) Adhesion of hAEC to AR-BP scaffolds (fibrous vs serous side). Each point represents mean of AlamarBlue intensity 6 h after seeding ± SD, n = 7 per seeding density per side (****p < 0.0001, *p < 0.05, for serous side). The presence of a basement membrane has no effect on hAEC adhesion in AR-BP scaffolds. (**B**) Proliferation of hAEC following seeding on AR-BP scaffolds. hAEC proliferation at 6 h post-seeding is significantly higher following seeding on the serous side versus the fibrous side. (**C**–**F**) hAEC proliferation following seeding at a density of (**D**) 71 cell/mm^2^; (**C**) 142 cell/mm^2^; (**E**) 284 cell/mm^2^; and (**F**) 568 cell/mm^2^, on the fibrous vs serous side of AR-BP scaffolds. Cell proliferation was significantly greater on the serous side than on the fibrous side at lower seeding densities.

Effect of scaffold ECM composition on hAEC behavior was investigated for AR-BP scaffolds. ECM niche had no impact on cell adhesion and viability 6 h post seeding (p > 0.05; Fig. 1A). Data from this experiment show that hAEC adhesion is not dependent on the side of AR-BP presented to them following seeding and increases linearly with seeding density up to 1,500 cell/mm^2^ (Supplemental Fig. S3).

Conversely, proliferation rate 6 h post-seeding at a moderate seeding density (142 cells/mm^2^) was significantly higher on the serous side (32 ± 5 cells per scaffold) than the fibrous side (14 ± 4 cells per scaffold) (p < 0.0001; Fig. 1B). Similarly, cell growth over time was affected by the ECM seeding side. At low and moderate seeding densities (71, 142 and 284 cells/mm^2^), proliferation of hAEC was significantly slower (Fig. 1C–E) on the fibrous side compare to the serous side (p < 0.0001 for 71 cells/mm^2^ and p < 0.05 for 142 and 284 cells/mm^2^), however at high density (568 cells/mm^2^) the effect of ECM niche on cell number was no longer detectable (Fig. 1F). No appreciable cell death was observed in either group.

### ECM niche modulates hAEC morphology

**Figure 2:**
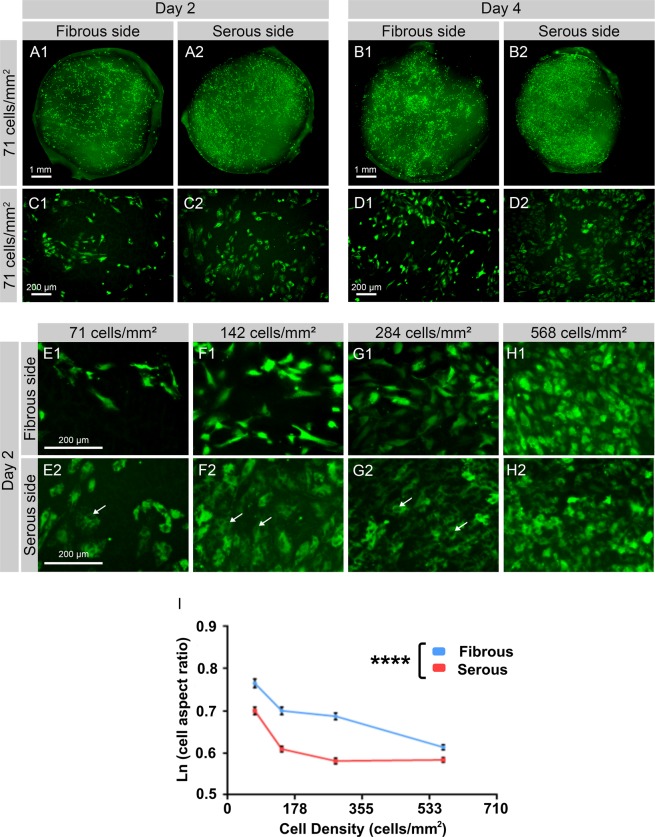
ECM niche modulates differential morphology of seeded hAEC. (**A**–**D**) Representative images of eGFP-hAEC seeded on fibrous or serous side of 6 mm AR-BP discs and imaged after 2 (**A**,**C**) and 4 (**B**,**D**) days show that higher number of cells can be observed on serous side compare to fibrous. (**E**–**H**) When seeded at higher densities and cultured for 2 days, eGFP-hAEC ((**E**) 71 cells/mm^2^; (**F**) 142 cells/mm^2^; (**G**) 284 cells/mm^2^, (**H**) 568 cells/mm^2^) seeded on the serous side show multiple vacuoles (arrows) and rounded morphology compared to fibrous side. (**I**) Quantification of cell aspect ratio demonstrates that cells seeded on serous side are statistically more rounded (lower aspect ratio) than those seeded on the fibrous side, and that cell morphology becomes increasingly more rounded with higher seeding densities regardless of seeding side. Scale bar 1 mm (**A**,**B**), 200 μm (**C**–**H**). Data represent the mean values natural log-transformed aspect ratios ± SD, n = 5 per group per seeding density (****p < 0.0001).

**Figure 3:**
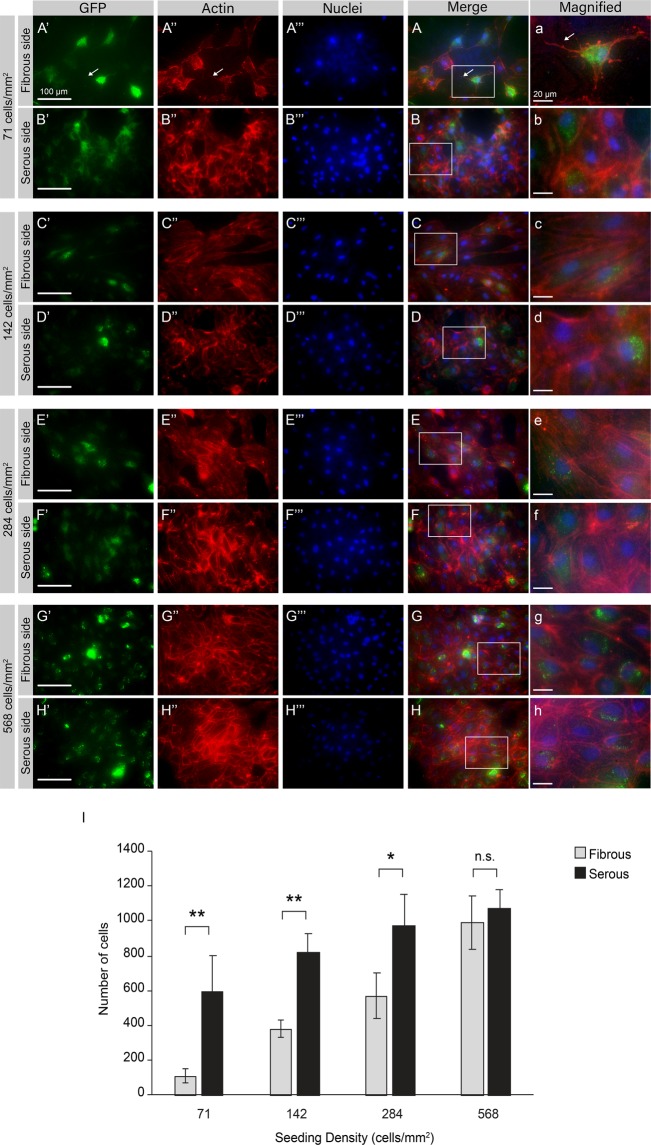
Actin expression in eGFP-hAEC seeded on AR-BP scaffolds. Representative microscopic images (n = 5 per group) of eGFP-hAEC (green, first column) after 4 days proliferation and staining for Actin (Phaliodin-594; red, second column) and nuclei (Hoechst 33342; blue, third column). (**A**,**B**) 71 cell/mm^2^; (**C**,**D**) 142 cell/mm^2^; (**E**,**F**) 284 cell/mm^2^ and (**G**,**H**) 568 cell/mm^2^. Scale bars: 100 μm (first to fourth column) and 20 μm (fifth column). (**I**) Quantification of cell number on the serous and the fibrous side 4 days after seeding at different densities demonstrate higher cell numbers in the serous side for low and moderate seeding densities (71, 142, and 284 cells/mm^2^), while cell number equalize when seeded at very high densities (568 cells/mm^2^). Data represent the average number of cells in a 1.6 mm^2^ area ± SD, n = 5 images per group (seeding density and side) (**p < 0.01, *p < 0.05, n.s. non-significant p = 0.4).

To determine the effect of ECM niche on endothelial cell morphology, fluorescent images of eGFP-hAEC seeded on serous vs fibrous sides of AR-BP scaffolds were taken on day 2 and day 4 after seeding. A greater number of cells were present on the serous side compared to fibrous (see Fig. 2A1,A2,B1,B2, and Supplemental Fig. S4 for qualitative assessment and Fig. 3I for cell number quantification), further supporting the cell growth results presented in Fig. 1B–E. Additionally, cells exhibited different morphology depending on the side of AR-BP they were seeded on (Fig. 2E-H). eGFP-hAEC seeded on serous side were more circular in shape and contained multiple vacuoles, whereas eGFP-hAEC on the fibrous side were more elongated and contained fewer vacuoles. This difference was not visible at high seeding density (568 cells/mm^2^) where the cells were completely confluent on both sides and had aspect ratios which were essentially circular, the characteristic cobblestone phenotype typical of endothelial cells (Fig. 2H1,H2). Morphological analysis quantified this finding, with cells seeded on the serous side having a statistically significantly lower, and thus more rounded, aspect ratio compared to those seeded on the fibrous side (Fig. 2I). Additionally, independent of seeding side there was a statistically significant decrease in cellular aspect ratio with higher seeding density.

Actin staining of cells following 4 days of culture on AR-BP scaffolds further illustrates the effect that the seeding side plays on endothelial morphology (Fig. 3A–H). At low seeding density hAEC on the fibrous side showed that cellular processes extend beyond the leading edge, with long spindly projections (white arrow, Fig. 3A”, Fig. 3A, and Fig. 3a for a magnified image). Additionally, cells exhibited a more elongated cell shape in the fibrous side than cells cultured on the serous side, especially at low to moderate seeding densities as seen in Fig. 3A/a vs 3B/b, 3C/c vs 3D/d, 3E/e vs 3F/f, in agreement with their measured higher aspect ratio (Fig. 2I). For cells cultured on the serous surface, a cortical actin arrangement was observed regardless of seeding density; however in cells seeded on the fibrous surface, at low seeding densities stress fibers were also seen along the entire cell in the orientation of the cell long axis. For higher seeding densities, fibrous seeding resulted in a change to more cortically arranged actin, similar to that observed for serous seeding.

### ECM modulates hAEC secretion of human laminin

**Figure 4:**
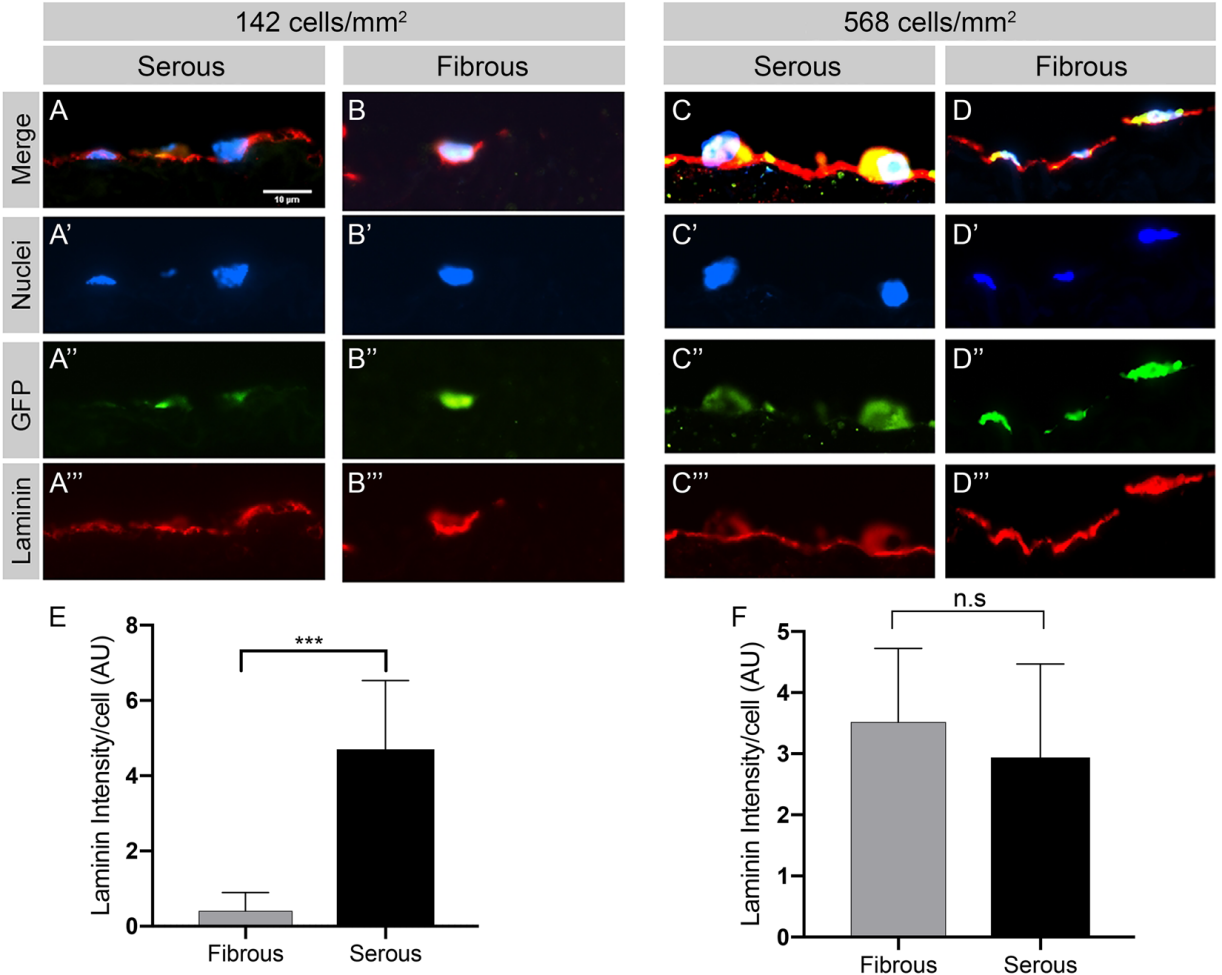
ECM niche modulates hAEC human laminin production following seeding on AR-BP scaffolds. Representative fluorescent microscopy images of (**A’**–**D’**) cell nuclei (blue), (**A”**–**D”**) eGFP-hAEC (green), and (**A”’**–**D”’**) human laminin (red). The serous side was found to have a continuous laminin layer while on the fibrous side new laminin production was found to be strongly associated with areas of cellular adhesion, especially at low seeding densities (n = 5 per group). (**E**,**F**) Quantification of human laminin production per cell. At moderate seeding density (142 cells/mm^2^) hAEC seeded on the serous side produce more laminin per cell than those seeded on the fibrous side (p = 0.0002). At high seeding density (568 cells/mm^2^) the effect of ECM niche on per cell laminin production is no longer present. Scale bar = 10 µm.

Immunofluorescence staining demonstrated preservation of native serous side basement membrane components (i.e., bovine laminin and collagen IV) in AR-BP scaffolds, whereas the fibrous side was devoid of such components^24^ (Supplemental Fig. S5). Human laminin deposition on the serous side formed a thin monolayer regardless of seeding density (Fig. 4A”’–D”’). Cells seeded on the fibrous side produced new human laminin concentrated within the local environment of each cell as observed from the fluorescence staining (Fig. 4A–D). New human laminin production on the fibrous side became increasingly confluent at higher seeding densities (Fig. 4B”’–D”’). Human laminin production per cell was higher on the serous side than on the fibrous side at moderate seeding densities (p = 0.0002; Fig. 4E). At high seeding densities, laminin production per cell was not significantly different between sides (p > 0.05; Fig. 4F).

### Short-term hAEC inflammation follows seeding on AR-BP

**Figure 5:**
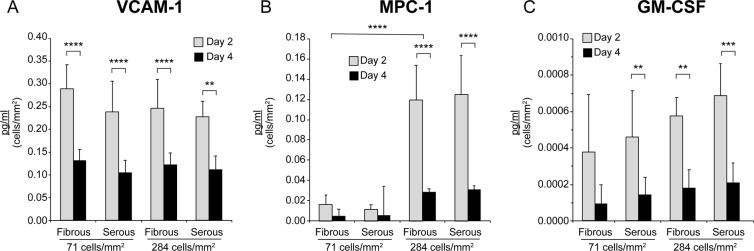
hAEC secretion of inflammatory markers decreases over time, especially at higher seeding densities but is not significantly impacted by ECM niche. Levels of (**A**) VCAM-1 are significantly lower on day 4 than on day 2. Similarly, secretion of inflammatory markers (**B**) MCP-1 and (**C**) GM-CSF tended to be lower on day 4 of culture. On day 2 MCP-1 levels are significantly higher at 284 cells//mm^2^ seeding density but were similar to lower seeding densities on day 4, while the other biomarkers remain unaffected by seeding density. Data are presented as the mean values of biomarker concentration normalized to cellular proliferation, ± SD, n = 7 (****p < 0.0001, ***p < 0.001, **p < 0.01).

Cytokine production and vascular cell adhesion marker (VCAM-1) levels were assessed to understand the intracellular processes that take place during hAEC culturing on AR-BP scaffolds (Fig. 5). Levels of VCAM-1 secreted to the culture media significantly decreased between day 2 and 4 on both the serous and fibrous side and at low and moderate seeding concentrations (71 and 284 cells/mm^2^), indicating a decrease in cellular inflammation over time (Fig. 5A). A similar effect was observed in monocyte chemoattractant protein-1 (MCP-1; Fig. 5B), and granulocyte macrophage colony-stimulating factor (GM-CSF; Fig. 5C), most prominently at moderate seeding density (284 cells/mm^2^). Intriguingly, MCP-1 levels at day 2 were significantly higher for hAEC seeded at 284 cells/mm^2^ than for cells seeded at 71 cell/mm^2^, though these levels decreased significantly by day 4, such that levels measured at low and moderate seeding densities showed no significant difference (Fig. 5B).

**Figure 6:**
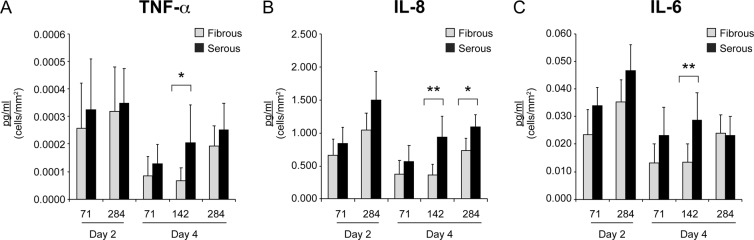
ECM niche modulates hAEC secretion of pro-inflammatory cytokines. Expression levels of (**A**) TNF-α, (**B**) IL-8, and (**C**) IL-6 exhibited an overall higher expression level on the serous side compared to the fibrous side of AR-BP scaffolds with significant increases observed after 4 days of culture at an initial seeding density of 142 cell/mm^2^. Each column represents concentration normalized to cell proliferation ± SD, n = 7; (**p < 0.01, *p < 0.05).

We further investigated whether the seeded side impacted the expression of the pro-inflammatory cytokines IL-8, TNF-α, and IL-6. A similar trend of decreasing inflammation with longer incubation times was observed, with the cells seeded on the serous side exhibiting an overall higher inflammatory response than those cultured on the fibrous side. At moderate seeding densities (142 cell/mm^2^), supernatant from cells at day 4 after seeding on serous side contained significantly higher levels of tumor necrosis factor alpha (TNF-α, Fig. 6A), interleukin 8 (IL-8, Fig. 6B) and IL-6 (Fig. 6C) than that from cells seeded on the fibrous side.

## Discussion

This study illustrates the impact that AR-BP scaffold fabrication parameters (i.e. complete removal of the cytotoxic antigen-removal solution) and sidedness (i.e., basement membrane vs. non-basement membrane ECM niche), have on endothelial adhesion, growth, phenotype, inflammatory response and laminin production. To allow for cellular attachment, an extended wash period was needed to ensure complete absence of the antigen-removal solutions following scaffold fabrication. Matrix niche, which changed based on which side of the AR-BP was presented to the cells during seeding, was found to impact cell proliferation rate, cellular morphology, human laminin production and had a moderate effect on pro-inflammatory cytokine production. In this study, the serous side resulted in faster hAEC proliferation, increased laminin production and rounded cell morphology, as well as an increase in the production of pro-inflammatory cytokines such as TNF-α, IL-6 and IL-8, for moderate seeding densities. Cell seeding density was found to impact the number of cells attaching to the scaffold, seeded cell growth, and some markers of the inflammatory response. At high seeding densities (i.e., near confluence) effects of matric niche on cell phenotype and function were no longer evident. The influence of ECM niche on cell responses at low and moderate seeding densities identified in this work has important potential implications for *in vivo* use of such acellular scaffolds in the early cellular repopulation timeframe (i.e., < 2 weeks), prior to endothelial monolayer formation.

Residual toxicity of implanted scaffolds is a critical consideration in the development of xenogeneic scaffolds, and has been one of the factors limiting their widespread adoption in clinical applications^32,33^. Here we demonstrate that hAEC are highly sensitive to the ASB-14 present in the antigen-removal solution, with ASB-14 showing cytotoxicity at relatively small concentrations (i.e., calculated LD_50_ 0.0021% w/v). This value is significantly lower than the previously reported LD_50_ for human mesenchymal stem cells (hMSC) of 0.0052% (w/v)^24^, suggesting that hAEC are more sensitive to ASB-14 toxicity than are hMSC. This assumption is supported by the fact that 6 days of washing were required in order for hAEC to grow on the AR scaffolds (Supplemental Fig. S1), whereas complete viability of hMSC seeded onto AR-BP scaffolds required only 4 days of washing^24^.

The surface of the AR-BP scaffolds seen by the cells is comprised of two distinct ECM niches, depending on the original anatomical orientation of the BP tissue: the fibrous side, which faces the thoracic cavity, and the serous side which faces the epicardium. Of these, the fibrous surface is comprised of a loose collagenous matrix, whereas the serous side exhibits a basement membrane containing proteins such as collagen IV and laminin, as well as proteoglycans^34^. The anisotropic organization of BP ECM niche results in sidedness which plays a critical role in the outcome of clinical applications utilizing this biomaterial. Previous studies have indicated that the presence or absence of basement membrane components plays an important role in modulating cellular adhesion and growth as well as matrix remodeling^34–37^. When used in vascular grafting procedures, the rough fibrous side exhibits a higher degree of adhesion to adjacent structures; and when turned to the inner portion of the vessel, a lower dilation compared to native tissue^38,39^. Conversely the serous side, with its intact basement membrane and complete laminin monolayer, exhibits lower acute cellular penetration into the vessel matrix, resulting in recellularization only on the scaffold surface^37,38^. In the current study, we demonstrated that both sides of the AR-BP scaffold retain an ECM composition suitable for endothelial adhesion with neither side exhibiting significantly different rates of cellular adhesion (Fig. 1). In contrast, cellular growth was significantly faster on the serous side, particularly at low and moderate seeding densities. These differences disappeared at high densities as the cells were seeded near confluence, limiting their ability to proliferate further. These differences in ECM modulated cell phenotype and function may have important clinical implications. If cellular repopulation and remodeling is of paramount importance, as in cardiovascular repair, the fibrous side could be utilized as it still demonstrates high cell binding and absence of a basement membrane on this side may allow for eventual cellular penetration into the collagenous matrix^24^. Conversely, if the formation of a monolayer is important, the presence of a basement membrane on the serous side would help to prevent cell penetration and fosters hAEC proliferation, resulting in the rapid formation of an endothelial monolayer. In addition to adhesion, basement membrane structures may influence cell signaling to the underlying ECM through modulation of integrin binding, while also influencing the signaling pathways that control cytokine expression^40^. These factors make the presence of an intact basement membrane a key consideration in determining the impact that AR-BP scaffold ECM niche will have on endothelial monolayer formation and new matrix production.

This study shows that AR-BP matrix niche also impacts endothelial cell morphology, with cells seeded on the serous side exhibiting the rounded, cobblestone characteristic morphology associated with healthy endothelium, even at low seeding densities. In contrast, cells seeded on the fibrous side exhibited an elongated morphology (Figs 2 and 3A–F), with long cellular projections and actin stress fiber presence observed at lower seeding densities (Fig. 3A). These morphological differences, combined with their faster cell growth, suggest hAEC seeded on the serous side would more rapidly form an intact endothelial monolayer compared to those seeded on the fibrous side, as we have seen in previous studies^41^. This endothelial layer would mask the underlying collagenous matrix, preventing platelet recognition and reducing acute thrombogenesis. Alternatively, serous side pre-seeding with endothelial cells would allow for the formation of an endothelial layer prior to implantation, reducing the incidence of thrombosis and intimal hyperplasia^42,43^, while increasing the patency of implanted vascular constructs as shown in previous studies^44,45^. Consequently, the effect of BP sidedness on hAEC morphology has implications for both acellular and seeded scaffold use in clinical practice.

Production of human laminin also differed with BP sidedness. The serous side of AR-BP scaffolds modulated rapid hAEC production of a thin intact human laminin layer, whereas the fibrous side exhibited a disrupted laminin layer which was associated predominantly with areas of cellular adhesion (Fig. 4). This suggests that adherent human aortic endothelial cells are locally synthesizing human laminin and that laminin production per cell is modulated by the ECM niche at moderate seeding densities^46,47^.

The data presented in this study indicate that AR-BP scaffold niche can have some impact on hAEC cytokine secretion (Figs 5, 6). We examined the expression of 5 cytokines (MCP-1, GM-CSF, TNF-α, IL-6, IL-8) and one cell adhesion molecule (VCAM-1) 2 and 4 days after seeding on different sides of the AR-BP scaffolds. We found that for most cytokines ECM niche did not play a significant role in regulating expression, with both serous and fibrous sides yielding similar levels of VCAM-1, MCP-1, IL-6, and GM-CSF. Of these, MCP-1 expression was found to be highly dependent on initial seeding density. As a marker for macrophage recruitment, MCP-1 upregulation would result in an increase in the presence of immune-presenting cells on the scaffold^48^. This suggests that endothelializing AR-BP scaffolds with high densities of hAEC may increase the immunogenicity of the resulting construct. Reassuringly, the expression levels of all measured cytokines and VCAM-1 decreased over time, with VCAM-1, MCP-1, and GC-CSF exhibiting significant decreases in expression levels between days 2 and 4 (Fig. 5). This trend indicates that elevated markers of inflammation including cytokine and VCAM-1 expression levels observed following seeding are the result of an acute inflammatory response which will decrease over time as the cells become confluent and enter a quiescent state. For pro-inflammatory cytokines IL-6, IL-8, and TNF-α a similar trend was observed, with expression levels decreasing from day 2 to 4 regardless of seeding side (Fig. 6). Interestingly, significant differences between expression levels were observed between serous and fibrous sides for TNF-α IL-6 and IL-8 at moderate densities, further indicating that cell density may play a role in modulating cellular response to ECM niche. This observation agrees with recent evidence of cell density impact on cytokines regulation and gene expression^49,50^. Further, TNF-α, IL-6 and IL-8 have been shown to upregulate laminin expression^48^, suggesting that their upregulation may be in response to basement membrane presence on the serous surface.

## Conclusion

There has been limited investigation into the effect that pericardial matrix sidedness has on the phenotype and function of seeded endothelial cells. In this study we demonstrate that basement membrane presence modulates hAEC laminin production, cell morphology and growth, and even exerts some effect on expression of pro-inflammatory markers at low and moderate seeding densities compared to seeding in the absence of basement membrane components (i.e., fibrous side). We found that seeding of hAEC onto the serous side of AR-BP scaffolds resulted in a more rounded cell morphology and a rapid cell growth compared to the fibrous side. Additionally, while cells seeded on both sides expressed similar levels of pro-inflammatory cytokines, this elevated expression appeared to be transient, with cytokine levels decreasing over time. The data presented in this study highlight the importance of considering matrix anisotropy when creating AR tissue scaffolds. When compared to previous studies that examined mesenchymal stem cell interactions with AR-BP scaffolds^24^ this study reports different trends in matrix toxicity, cell morphology, and cytokine expression, suggesting that further studies with other cell types of interest (i.e. smooth muscle cells) are warranted.

## Materials and Methods

### Human aortic endothelial cell culture and expansion

Human aortic endothelial cells (hAEC) were chosen as an EC model for their relevance in cardiovascular function and disease^51,52^. Enhanced green fluorescent protein (eGFP) transfected hAEC were purchased from Angio-Proteomie (Boston, MA) (CAT No. cAP‐0006GFP, LOT No. 201209701SR). Cells were cultured according to manufacturer protocol. Briefly, eGFP-hAEC were thawed, suspended in complete EGM-2 media (Lonza, Walkersville, MD) supplemented with 10% fetal bovine serum (FBS) (Atlanta Biologicals, Lawrenceville, GA) (EGM-2F) and plated in a T-75 flask pre-coated with quick coating solution (Angio-Proteomie). All cells were cultured at 37 °C and 5% CO_2_, and upon reaching 80% confluency, were passaged at a 1:4 ratio. All experiments utilized cells from passage 4–6 (P4-P6).

Human AECs were purchased from Lonza (Walkersville, MD), (CAT No. CC-2535, LOT No 0000303583) and cultured in the same way as described for eGFP-hAEC.

### Fabrication of AR-BP discs

Bovine pericardia (BP) were harvested from young adult cattle (Spear Products, Coopersburg, PA), epicardial fat was removed and 1 cm wide strips were stored in storage solution containing 15% (v/v) dimethyl sulfoxide (DMSO) and 85% Dulbecco’s Modified Eagle Media (DMEM, Sigma, St Louis, MO) at −80 °C. Strips were dissected into approximately 1 cm × 1 cm intact pieces (0.18–0.22 g) and subjected to an AR protocol as previously described^23^. Briefly, pieces were incubated with hydrophile solubilization buffer (100 mM DTT, 2 mM MgCl_2_-6H_2_O, 100 mM KCl, 0.5 mM Pefabloc and 1% (v/v) AAS (antibiotic antimycotic solution, Sigma, St Louis, MO) in 10 mM Tris-HCl, pH 8.0) for 48 h at 4 °C, followed by lipophile solubilization using 1% (w/v) amidosulfobetaine-14 (ASB-14, Sigma, St. Louis, MO) dissolved in hydrophile solubilization buffer for 48 h at room temperature (RT), 24 h of nuclease treatment (2.5 Kunitz units/mL DNAse I, 7.5 Kunitz units/mL RNAse A, 1% (v/v) AAS, 0.15 M NaCl, 5 mM MgCl_2_-6H_2_O in 10 mM Tris-HCl, pH 7.6) and wash out using 0.5 mM Pefabloc and 1% (v/v) AAS in 1x Tris-Buffered saline for 144 h. Antigen removed BP pieces were then placed in storage solution at −80 °C. To ensure sterility, all solutions were sterile filtered prior to use and all solution changes were performed aseptically.

For creation of AR-BP discs for cell culture, antigen removed pieces were incubated overnight in EGM-2F cell media at RT and then washed in fresh cell media for additional 15 min. Discs were generated from tissue pieces using 6 mm biopsy punches (Acuderm Inc., Fort Lauderdale, FL), placed in a 96-well plate and seeded with eGFP-hAEC suspended in 200 µL of media, for a final seeding density of 71 to 3537 cells/mm^2^. To allow for direct comparison, fibrous and serous discs were always generated from the same piece of antigen removed BP. BP pieces were asymmetric and physically marked during the first steps of preparation in order to allow unambiguous identification and tracking of sidedness. For cell culture experiments, biopsy sizes were chosen to closely approximate well size, thereby ensuring that samples were unable to flip during the cell culture period. Additionally, all cell seeding, media changes, and rinses were done very slowly to avoid inadvertent flipping of the material during manipulations.

### Cytotoxicity assay

MultiTox-Fluor multiplex cytotoxicity assay (Promega, Madison, WI) was used to determine the toxicity of ASB-14 to hAEC. Cells were seeded on tissue culture treated 96-well plate (Corning, Corning, NY) at 167 cell/mm^2^ concentration. Following overnight culture, cells were incubated with fresh EGM-2F media for 1 h (to remove built up metabolites). Cells were then incubated with ASB-14 (0.012%, 0.0070%, 0.005%, 0.035%, 0.002% w/v) dissolved in 100 µl of culture media for 1 h at 37 °C, 5% CO_2_. Following incubation with spiked media, cells were incubated with 100 µl of MultiTox-Fluorfor Reagent for 1.5 h at 37 °C, 5% CO_2_. Fluorescence intensity was measured using Cytation3 (BioTek, Winooski, VT) plate reader, with live (400Ex/505Em nm) and dead (485Ex/520Em nm) cell fluorescence measured. Fluorescence ratio was calculated as relative fluorescence units (RFU) of live cells/RFU dead cells in the same well. Percentage of viable cells was calculated as fluorescence ratio of the sample/fluorescence ratio of 100% live cells (untreated cells). 60 µg/mL digitonin was used as 100% dead control. All data points were generated from the mean of technical triplicates, with N = 6 experimental replicates for each detergent concentration.

### Cell proliferation Assay

hAECs (p2) were plated in a six well plate at a density of 100,000 cells per well. The cells were transfected over night with 150 µL of Premo FUCCI cell Cycle Sensor (Thermo Scientific, Waltham, MA) in 700 µL of EGM-2 cell media (Lonza, Walkersville, MD). After overnight incubation, cells were lifted using Accutase (DMEM, Sigma, St Louis, MO) and seeded on 14 mm BP-AR scaffolds at a density of 142 cells/mm^2^. Six hours after seeding proliferating cells per scaffold were counted using a Nikon Eclipse Ni-E microscope (Nikon, Melville, NY).

### AlamarBlue assay

AlamarBlue® Assay (Invitrogen, Carlsbad, CA) was utilized to monitor hAEC proliferation following seeding. eGFP-hAEC were seeded at 37 °C, 5% CO_2_ on the fibrous or serous surface of 6 mm AR-BP discs, with tissue culture treated plastic cells utilized as control. At each time point, cells media was replaced with 120 μL fresh culture media and 12 μL AlamarBlue reagent. After 1.5 h of incubation at 37 °C, 5% CO_2_, 100 µL of solution from each well was transferred to a 96-well plate for measurements. Fluorescence intensity (560Ex/590Em nm) was measured using SpectraMax M5 (Molecular Devices Sunnyvale, CA) plate reader, with solution from wells without cells serving as background fluorescence. For longitudinal studies, all residues of almarBlue reagent were removed from the wells and 250 µl of fresh media were added. N = 6 experimental replicates per group per time point.

### Residual BP scaffold cytotoxicity

During the AR protocol, washout buffer was collected (1, 2, 3, 4, 6 days) and stored at −20 °C (n = 8 per group) for cytotoxicity evaluation. eGFP-hAEC were seeded on tissue culture treated 96-well plate (Corning, Corning, NY) at 222 cell/mm^2^ concentration. After overnight culturing at 37 °C, 5% CO_2_, cells were washed with fresh EGM-2 media (no extra FBS) to remove built up metabolites and then incubated with EGM-2 media spiked with 40% washout buffer for 3 h at 37 °C, 5% CO_2_. AlmarBlue analysis was performed as described above. The results were normalized to a reading from cells cultured in 60% EGM-2, 40% wash buffer. Culture media served as background fluorescence. All data points were generated from the mean of technical triplicates, with N = 8 experimental replicates for each detergent concentration.

### Cells seeding and imaging on antigen removed scaffold

AR-BP discs were placed in a 96-well plate and seeded with eGFP-hAEC suspended in endothelial media for a final seeding density of 69 to 3537 cells/mm^2^. Fibrous and serous discs were always generated from the same piece of AR-BP. To determine cell adhesion rate, cells were seeded on the serous or fibrous side of AR-BP discs and incubated for 6 h. Seeding media was removed, samples were rinsed of unbound cells, and an AlamarBlue assay was performed to quantify cellular adhesion. For proliferation studies seeded cells (71, 142, 284, 568 cells/ mm^2^) were cultured for 24 h prior to measurement with AlamarBlue. Proliferation measurements were repeated on days 2 and 4. For cell size and morphology analysis, cells were cultured for 48 or 96 h at 37 °C, 5% CO_2_ before fluorescent imaging using a BZ-X710 fluorescence microscope (Keyence, Itasca. IL). Images were acquired with 4x and 10x objectives and XY or Z-Stack planes (maximum contrast projection) were combined using BZ-X analyzer. Sets of 12–15 images were analyzed per group (seeding density and sidedness, total of 10 groups) using MATLAB (MATLAB ®, The MathWorks, Inc., Natick, Massachusetts, United States). All cells in each image were measured to quantify major and minor cell axis length, and major to minor cell axis ratio was calculated. All length and aspect ratio data were normalized using natural log transformation prior to analysis. Consequently, a value of 0 represents a perfectly circular aspect ratio.

### Immunofluorescence staining

For immunofluorescence, cell seeded scaffolds were washed with DPBS (HyClone, South Logan, Utah), fixed with 4% formaldehyde (Thermo Scientific, Waltham, MA) for 40 min, washed with DPBS and incubated with 0.1% Triton x-100 with 1% BSA for 10 min. After washing with PBS, Acti-stain-594 Phalloidin (1:40) and Hoechst 33342 (1:10,000) (Invitrogen, Carlsbad, CA) were added for 30 min. Following washing with PBS, scaffolds were immersed in ProLong Gold antifade reagent (Invitrogen, Carlsbad, CA) and placed on a microscope slide using Gene Frame as space keeper (Thermo Scientific, Waltham, MA). Samples were imaged using BZ-X710 Fluorescent microscope (Keyence, Itasca. IL) using 10x and 40x objectives and XY or Z-stack planes were combined using BZ-X Analyzer software.

For histological imaging of cellular location and laminin production, BP scaffolds seeded with GFP-labeled hAECs were subjected to triple immunofluorescence staining on day 4 after seeding. Briefly, the scaffolds were fixed in 10% buffered formalin, embedded in paraffin and 4 µm sections mounted on glass slides. Immunofluorescence triple staining was performed using mouse anti-eGFP and human laminin stained with rabbit anti-laminin primary antibodies (1:20, 1:200 respectively, Abcam, Cambridge, MA). Fluorescent anti-mouse secondary antibody (1:200, Invitrogen) tagged with Alexa Fluor 546, anti-rabbit secondary antibody tagged with Alexa Fluor 647 (1:200, Abcam) and DAPI (ProLongTM Gold antifade reagent with DAPI, Invitrogen) were used for visualization. Collagen IV was stained using a rabbit anti-collagen IV antibody (1:200, Abcam, Cambridge, MA). Bovine Laminin was stained using a rabbit anti-laminin antibody (1:20, Invitrogen, Carlsbad, CA). Fluorescent anti-rabbit secondary antibody tagged with Alexa Fluor 647 (1:200, Abcam, Cambridge, MA) and DAPI (ProLongTM Gold antifade reagent with DAPI, Invitrogen) were used for visualization. For all stains, slides were imaged using a Nikon Eclipse E600 microscope and digital images collected.

For human laminin quantification, 20x images were analyzed using MATLAB (MATLAB ®, The MathWorks, Inc., Natick, Massachusetts, United States) (n = 6 scaffolds per group). Total human laminin fluorescence was quantified for each image, with laminin per cell calculated by divided by the number of cells present based on counting of DAPI stained nuclei in the same imaging field.

### Biomarkers analysis

AR-BP scaffolds seeded on either the fibrous or serous side with eGFP-hAEC (71, 142, 284 cells/mm^2^) scaffolds were cultured in EGM-2F media, with media changed every day (n = 7 per group, per seeding concentration). After 48 and 96 h, supernatant media was collected and stored in −80 °C. AlmarBlue assay was performed at each supernatant collection timepoint and data used for normalization in subsequent experiments.

Collected media from each scaffold (n = 7 per group, per seeding concentration) was assayed using V-PLEX MULTI-SPOT assay system (Meso Scale Diagnostics, Rockville, MD) according to the manufacturer’s instructions. Assays were performed for IL-6, IL-8 and TNF-α in the pro-inflammatory panel, human GM-CSF and human IL-1α in the cytokines panel, human MCP-1 in the chemokines panel and human VCAM-1 in the human vascular injury panel. Array data were measured on a MSD QuickPlex SQ 120 (Meso Scale Diagnostics, Rockville, MD) and analyzed by MSD discovery workbench 4.0.12. Biomarkers concentrations were determined by comparing data to standards obtained with test kits. Obtained concentrations were normalized to AlamarBlue data obtained following supernatant collection.

### Statistical analysis

To determine cytotoxicity of residual detergents in scaffolds one-way ANOVA followed by Tukey’s multiple comparisons post hoc test was performed. For cell adhesion to the scaffolds two-way ANOVA analysis and Tukey’s multiple comparisons post hoc tests were performed. To compare proliferation two-way ANOVA analysis with repeated measurements was performed. For biomarker analysis between days 2 and 4 repeated measurements three-way ANOVA analysis and Tukey’s multiple comparisons post hoc tests were performed. These data were expressed as mean ± standard deviation (SD) and p < 0.05 was considered to be significant. For sidedness effect on biomarker on day 4, two-way ANOVA analysis and Tukey’s multiple comparisons post hoc tests were performed. Aspect ratio comparisons were done with two-way ANOVA and Tukey’s multiple comparisons post hoc tests. These data were expressed as mean ± standard error of the mean and p < 0.05 was considered to be significant.

## Supplementary information

Supplementary Information

## Data Availability

The datasets generated and analyzed during the current study are available from the corresponding author on reasonable request.
