# Supplementary material for: Bovine pericardial extracellular matrix niche modulates human aortic endothelial cell phenotype and function
Source: Sci Rep. 2019 Nov 13;9:16688. doi: 10.1038/s41598-019-53230-1 (PMC6853938; doi:10.1038/s41598-019-53230-1)

**Bovine pericardial extracellular matrix niche modulates human aortic endothelial cell phenotype and function**

Jeny Shklover^a,b^, James McMasters^b^, Alba Alfonso-Garcia^b^, Manuela Lopera Higuita^c^, Alyssa Panitch^b^, Laura Marcu^b^, Leigh Griffiths^c,^ *

^a^ Department of Chemical Engineering, Israel Institute of Technology, Haifa 31096, Israel (current affiliation)

^b^ Department of Biomedical Engineering, University of California Davis, One Shields Avenue, Davis, CA 95616, United States

^c^ Department of Cardiovascular Diseases, Mayo Clinic, 200 First St. SW, Rochester, MN 55905, United States

* Corresponding Author: [griffiths.leigh@mayo.edu](mailto:griffiths.leigh@mayo.edu)

**Supplementary Information**

**Figure S1.** **Residual ASB-14 toxicity is removed from the scaffold following 6 days of washing.** (A) Relative toxicity of ASB-14. Each point represents mean ± standard deviation (SD) of % viable cells after incubation with different concentration of ASB-14, n = 6 per group and time point. Lethal dose of ASB-14 resulting in 50% cell death (LD50) calculated to be 0.0021% w/v. (B) Analysis of the washout solution on days 1-6 to determine scaffolds’ residual toxicity to hAEC. n = 8; Residual toxicity of ASB-14 treated scaffolds reaches 0% following 6 days of washing (****p<0.0001, **p=0.0051, *p=0.0112) (C-E) hAEC-GFP growth on native BP (C) versus AR-BP scaffolds washed for 4 (D) or 6 (E) days. Cell adhesion and growth is reduced at 4 days of washing but reaches control levels following 6 days of washing. Scale bar 100 μm.


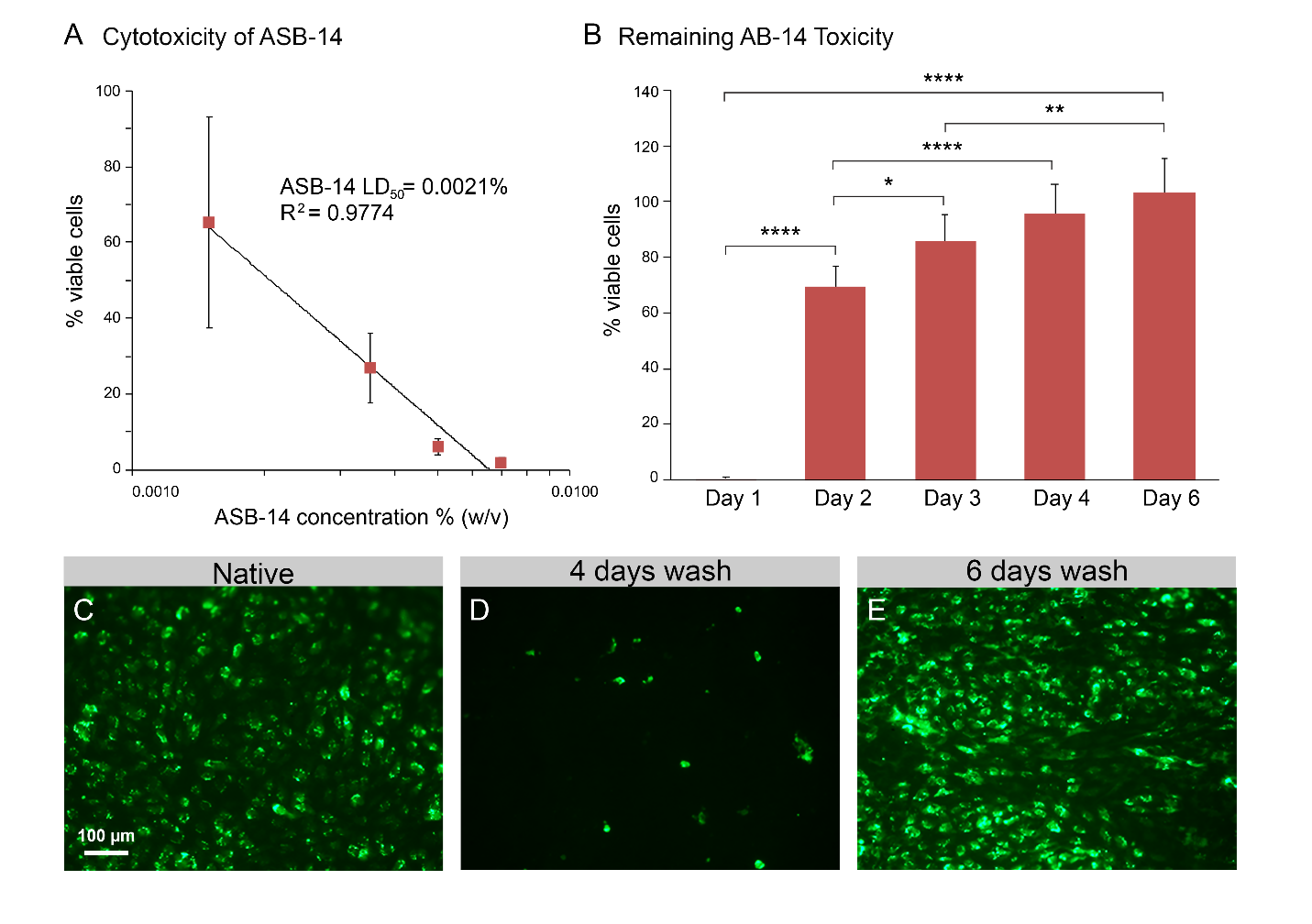


**Figure S2. Residual ASB-14 toxicity is removed from the scaffold following 6 days of washing.** (A) After 4 days, samples washed in cell culture media for 4 hours exhibited reduced cellular attachment compared to those washed for 6 days. (B) Cellular attachment of cells seeded on different sides of the AR-BP showed similar trends, with 6 days of washing being required for robust cellular attachment.


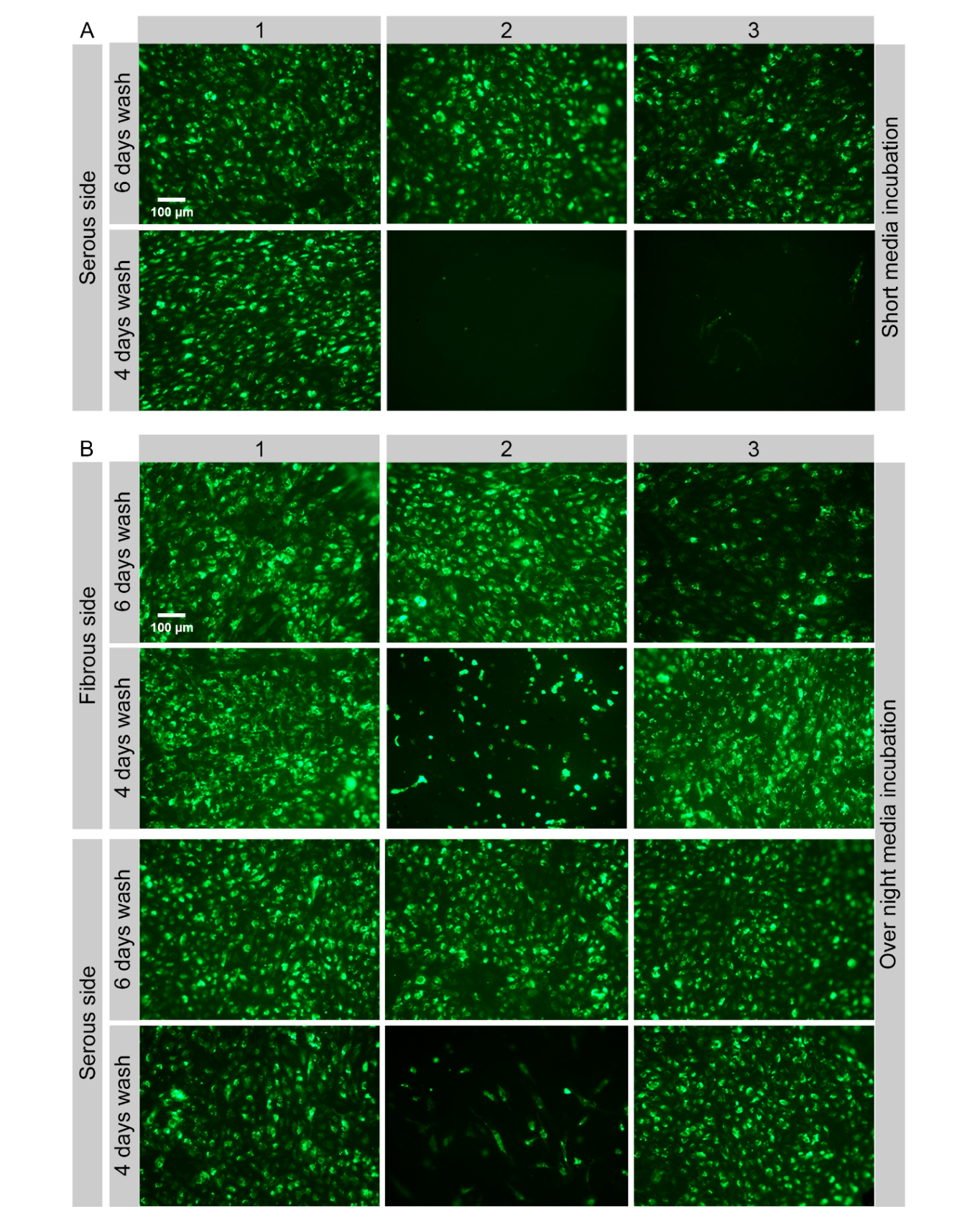


**Figure S3. Cellular attachment is linear at low seeding densities.** (A) Endothelial attachment becomes non-linear at seeding densities above 1500 cells/mm^2^ (B) while attachment remains linear at lower seeding densities, suggesting cells are being seeded at near confluence at densities approaching 1500 cells/mm^2^.


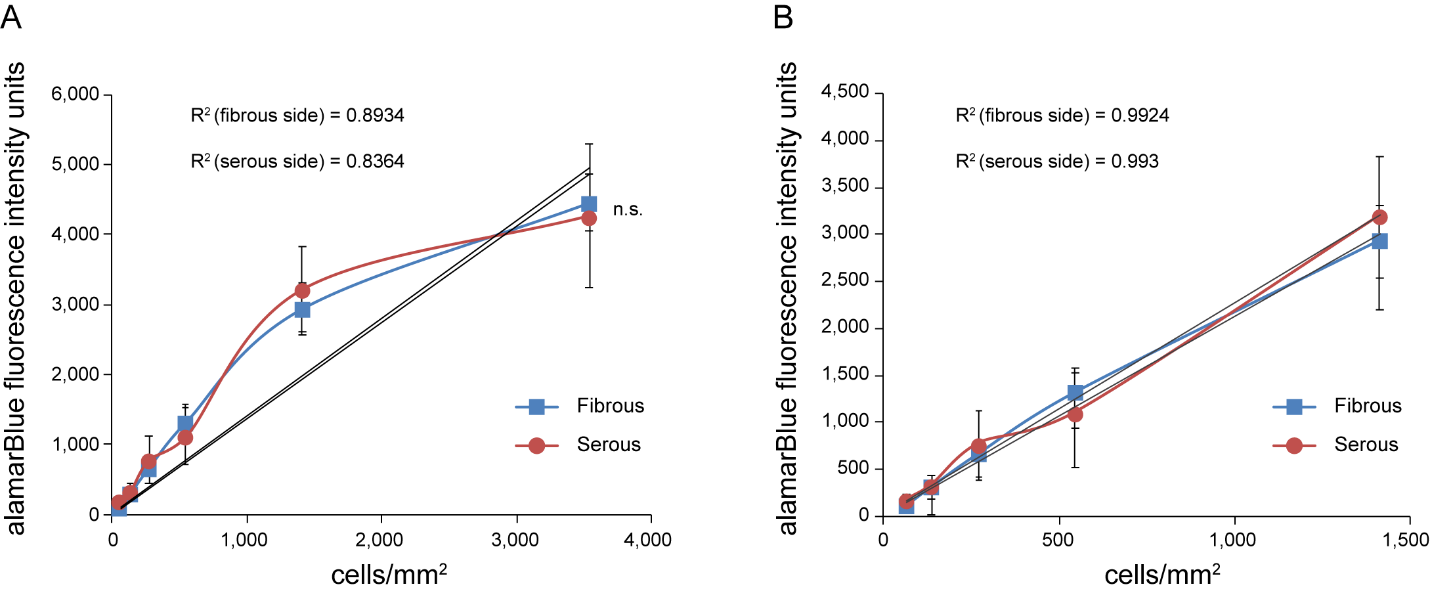


**Figure S4. Cellular proliferation increases with initial seeding density.** (A) After 2 days the serous side exhibits faster cellular proliferation at seeding densities below 568 cells/mm^2^. (B) This trend continues after 4 days of culturing, with cellular density on the serous side approaching confluence at seeding densities above 71 cells/mm^2^ at both 2 and 4 days of culturing.


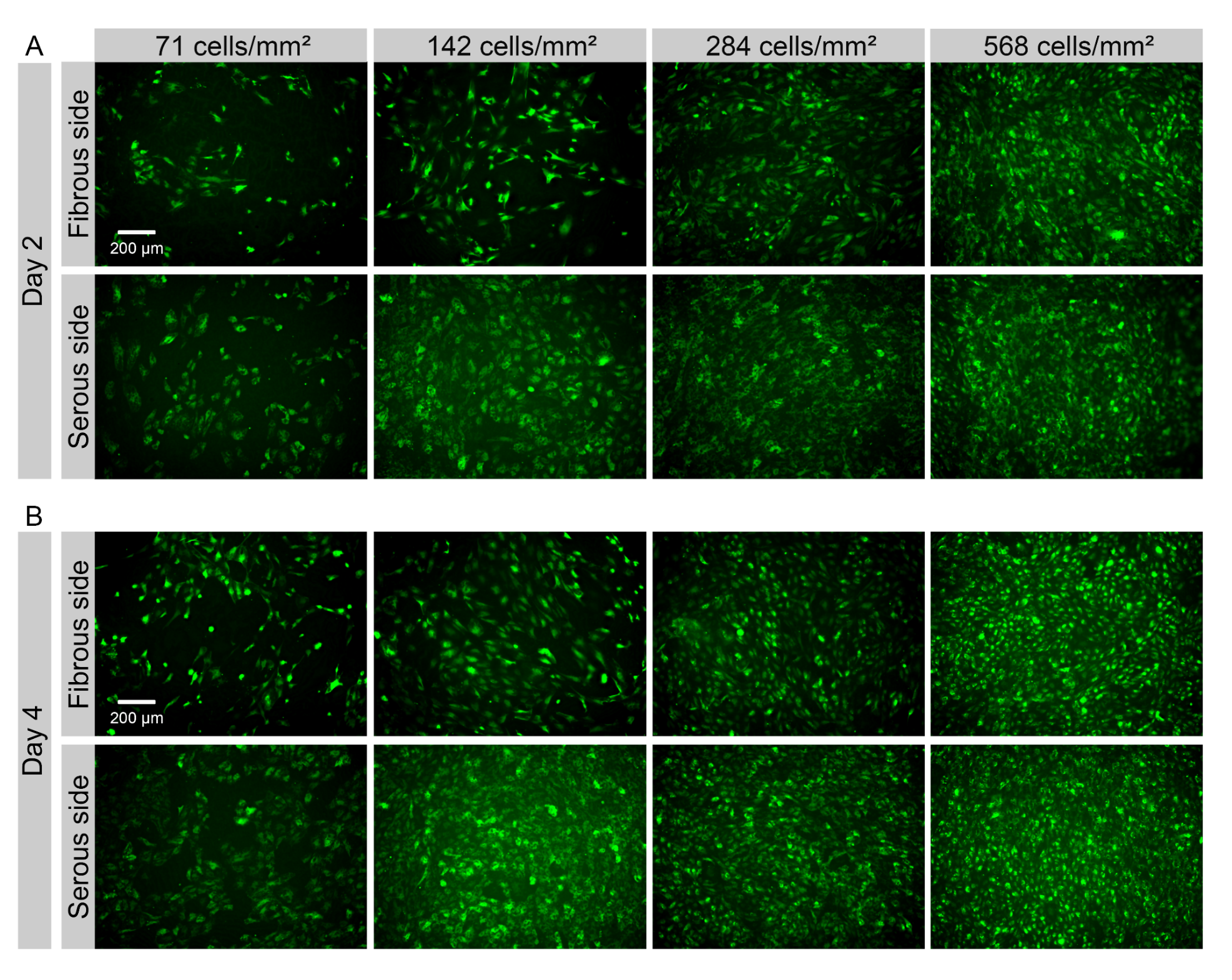


**Figure S5. Protein staining of the AR-BP extracellular matrix.** Native bovine laminin staining in AR-BP serous (A) and fibrous side (B) show laminin presence in the basement membrane of the serous side and lack thereof in fibrous side. Staining for human laminin in the serous (C) and the fibrous (D) sides demonstrate this antibody does not cross-react with the bovine laminin present in the AR-BP scaffold. Native bovine collagen IV staining in AR-BP serous (E) and fibrous side (F) further characterize the unique structural composition of the basement membrane.


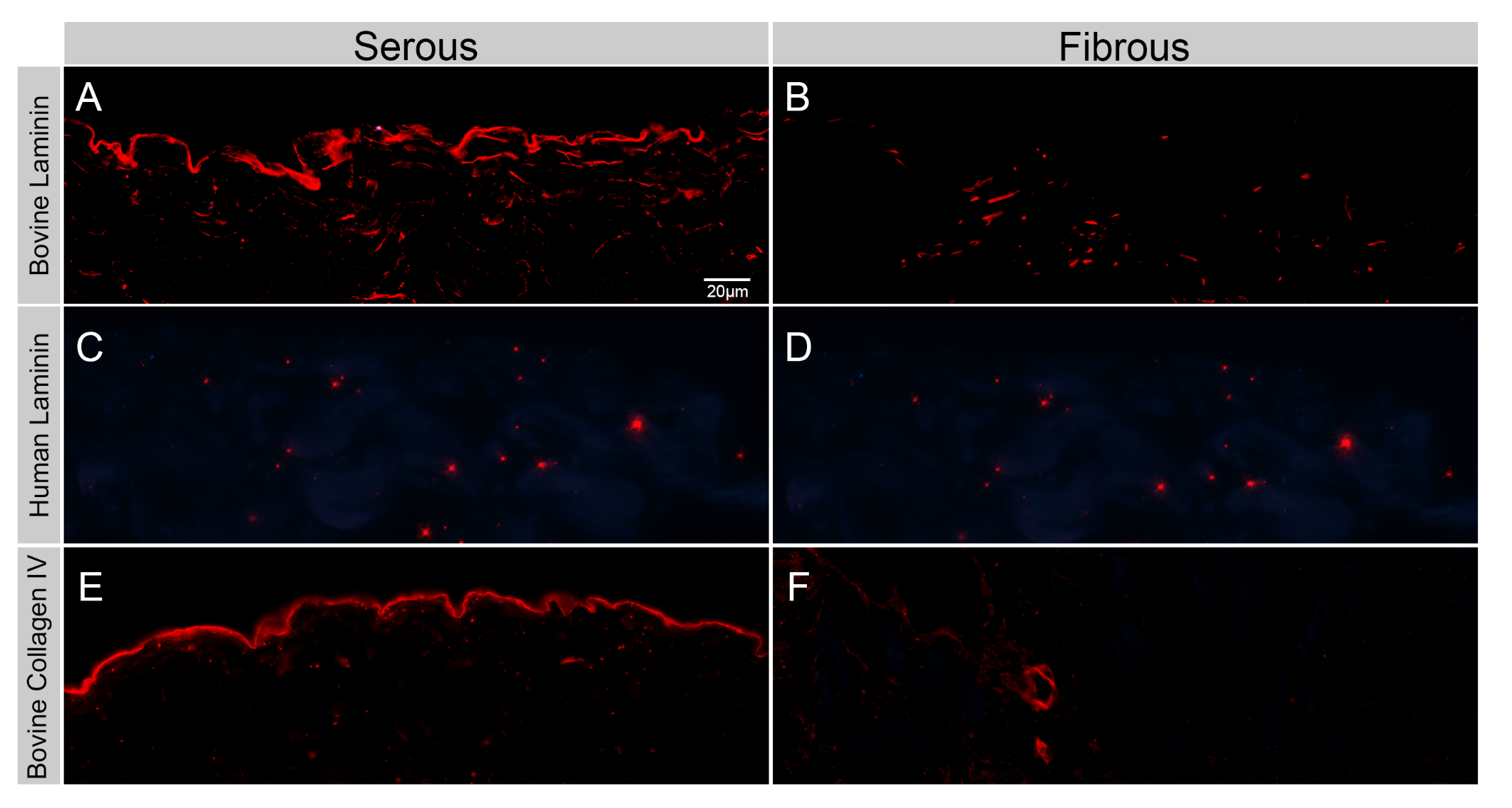

Supplement: Supplementary file 1 — Supplementary Information [file 41598_2019_53230_MOESM1_ESM.docx]
